# A Phase II, Randomized, Double-blind, Controlled Safety and Immunogenicity Trial of Typhoid Conjugate Vaccine in Children Under 2 Years of Age in Ouagadougou, Burkina Faso: A Methods Paper

**DOI:** 10.1093/cid/ciy1104

**Published:** 2019-03-07

**Authors:** Matthew B Laurens, Sodiomon B Sirima, Elizabeth T Rotrosen, Mohamadou Siribie, Alfred Tiono, Alphonse Ouedraogo, Yuanyuan Liang, Leslie P Jamka, Karen L Kotloff, Kathleen M Neuzil

**Affiliations:** 1 Center for Vaccine Development and Global Health at the University of Maryland School of Medicine, Baltimore, MD; 2 Groupe de Recherche Action en Santé, Ouagadougou, Burkina Faso; 3 Department of Epidemiology and Public Health University of Maryland School of Medicine, Baltimore, MD

**Keywords:** child, typhoid-paratyphoid vaccines, Burkina Faso, immunogenicity, conjugate

## Abstract

The recent Typhoid Fever Surveillance in Africa Program demonstrated an overall adjusted incidence of typhoid fever 2–3 times higher than previous estimates in Africa. Recently, a single-dose typhoid conjugate vaccine that allows infants as young as 6 months old to be vaccinated was prequalified by the World Health Organization (WHO). This Vi-based conjugate vaccine demonstrated robust immunogenicity after 1 dose in infants and children 6 through 23 months of age in India with no safety signal, and is currently being tested for the first time on the African continent in Malawi. The WHO Strategic Advisory Group of Experts recommends studies to evaluate co-administering Vi-typhoid conjugate vaccine (Vi-TCV) with routine childhood vaccines in typhoid-endemic countries. The Burkina Faso immunization schedule includes yellow fever vaccine (YFV) at 9 months and meningococcal A conjugate vaccine (MCV-A) at 15 months, in addition to measles-rubella vaccine at both 9 and 15 months. Co-administration testing of Vi-TCV with these routine vaccinations will provide the data needed to support large-scale uptake of Vi-TCV in sub-Saharan Africa. A randomized, controlled, Phase II trial of Vi-TCV co-administration with the vaccinations routinely given at 9 and 15 months of age is planned in Burkina Faso. The overall aim is to assess the safety and immunogenicity of Vi-TCV when co-administered with YFV at 9 months of age and with MCV-A at 15 months of age. A total of 250 participants (100 infants aged 9–11 months and 150 children aged 15–23 months) will be enrolled.

***Clinical Trials Registration.*** NCT03614533.

Estimating the typhoid fever incidence is challenging in low- and middle-income countries, due to the non-specific clinical disease presentation and limited access to blood cultures, the most reliable laboratory diagnostic. The recent Typhoid Fever Surveillance in Africa Program measured the incidence of invasive *Salmonella* bloodstream infections in 10 African countries over a 27-month period [1] and demonstrated an overall adjusted incidence of typhoid fever 2–3 times higher than previous estimates [2]. Importantly, 47% of *Salmonella enterica* serovar Typhi isolates were multidrug resistant. Similar studies have documented a significant burden of typhoid fever in sub-Saharan Africa [3–6]. In Burkina Faso, the adjusted incidence of *S*. Typhi ranged from 104 to 383 per 100 000 person-years of observation (PYO) for all ages across 2 study sites. The highest adjusted incidence occurred in children 5–14 years of age at Nioko II (315 per 100 000 PYO) and in children 2–4 years of age at Polesgo (1890 per 100 000 PYO) [1].

In addition to clean water and adequate sanitation, the World Health Organization (WHO) recommends vaccination against typhoid fever as an effective preventive measure. Before 2017, only 2 typhoid vaccines were available [7]: (1) the oral, live-attenuated Ty21a vaccine, licensed for children ≥6 years of age, and (2) an injectable Vi capsular polysaccharide vaccine, licensed for children ≥2 years of age. Recently, Bharat Biotech International developed a conjugate vaccine (Typbar-TCV) with Vi polysaccharide conjugated to a nontoxic tetanus toxoid (TT) protein carrier. This Vi-typhoid conjugate vaccine (Vi-TCV) stimulates both a strong, T-cell–dependent response and a more robust, anti-Vi response as compared to an unconjugated Vi polysaccharide vaccine. Vi-TCV safety and immunogenicity have been studied in adults and children as young as 6 months of age in India [8], where a single dose elicited 4-fold seroconversion rates of 98% and 99% in participants 6–23 months and 2–15 years, respectively [8]. This vaccine holds significant promise, because of its single-dose schedule and utility in infants as young as 6 months of age.

While Vi-TCVs have been extensively studied in Asia, an ongoing clinical trial of Typbar-TCV in Malawian infants and children is the first-ever trial of any Vi-TCV in Africa [9]. This study will provide valuable data to sub-Saharan African decision-makers. However, because Expanded Program on Immunization (EPI) schedules differ throughout the continent, the WHO Strategic Advisory Group of Experts recommends studies to evaluate co-administering Vi-TCV with routine childhood vaccines in typhoid-endemic countries.

Available data from Asia on Typbar-TCV co-administration with measles-containing vaccines shows no altered immune response to either product; this result is being confirmed in Malawi in an immunogenicity sub-study of measles-rubella vaccine (MR) co-administration with Typbar-TCV [9]. Co-administration studies with yellow fever vaccine (YFV) and meningococcal A conjugate vaccine (MCV-A) are needed. Burkina Faso is the ideal study site, given its elevated burden of typhoid fever, elevated infant mortality, high malaria prevalence, ongoing typhoid surveillance, existing research infrastructure, and history of early introduction of WHO pre-qualified vaccines. The Burkina Faso immunization schedule includes YFV at 9 months and MCV-A at 15 months, in addition to MR at both 9 and 15 months.

## TRIAL OVERVIEW

A randomized, controlled, Phase II trial will test Vi-TCV co-administration with vaccinations routinely given at 9 and 15 months of age in Burkina Faso. Our aim is to assess the safety and immunogenicity of Vi-TCV when co-administered with vaccinations routinely given to children at both 9 and 15 months of age in Burkina Faso. The Vi-TCV vaccine or an inactivated poliovirus vaccine (IPV) will be administered. Children 9–11 months of age will be randomized 1:1 to Vi-TCV or IPV; children 15–23 months of age will be randomized 1:1:1 to Vi-TCV + MCV-A, Vi-TCV + IPV with delayed MCV-A, or MCV-A + IPV. We will use block randomization with varying block sizes from 6–12. Our study objectives and endpoints are listed in Table 1.

**Table 1.** Primary, Secondary, and Exploratory Objectives and Endpoints

	Objectives	Endpoints
**Primary**	To assess the safety of Vi-TCV when co-administered with Expanded Program on Immunization vaccines among children 9–11 months of age and 15–23 months of age in Burkina Faso.	1. Proportion of participants who develop adverse events in the first 30 minutes after vaccination and for 7 days after vaccination. 2. Proportion of participants who experience SAEs within 6 months of vaccination, in all participants. 3. Proportion of participants who experience other, non-serious adverse events up to 28 days following vaccination.
**Secondary**	1. To assess the immunogenicity of YFV when given with and without Vi-TCV in children 9–11 months of age. 2. To assess the immunogenicity of MCV-A when given with and without Vi-TCV in children 15–23 months of age. 3. To assess the immunogenicity of Vi-TCV when given with YFV in children 9–11 months of age. 4. To assess the immunogenicity of Vi-TCV when given with and without MCV-A in children 15–23 months of age.	1. Immunogenicity of YFV: yellow fever plaque reduction neutralization test at Days 0 and 28 in infants 9–11 months of age. 2. Immunogenicity of MCV-A: serum bactericidal antibody assay against *Neisseria meningitidis* serogroup A at Days 0 and 28 in children 15–23 months of age. 3. Immunogenicity of Vi-TCV when given with YFV: anti-Vi IgG antibody titer, as measured by ELISA at Days 0 and 28 in infants 9–11 months of age. 4. Immunogenicity of Vi-TCV when given with and without MCV-A: anti-Vi IgG antibody titer, as measured by ELISA at Days 0 and 28 in infants 9–11 months of age.
**Exploratory**	1. To assess the TT IgG antibody levels in children of all ages who receive any vaccine regimen. 2. To assess whether the presence of *P. falciparum* parasitemia is associated with a decreased immunogenic response to vaccination with YFV, Vi-TCV, TT, and MCV-A.	1. Anti-TT IgG antibody level in all children who receive any vaccine regimen. 2. Immunogenicity of YFV, Vi-TCV, TT, and MCV-A in vaccinated children with and without *Plasmodium falciparum* parasitemia present at vaccination.

Abbreviations: ELISA, enzyme-linked immunosorbent assay; IgG, immunoglobulin G; MCV-A, meningococcal A conjugate vaccine; SAE, serious adverse event; TT, tetanus toxoid; Vi-TCV, Vi-typhoid conjugate vaccine; YFV, yellow fever vaccine.

Following the routine vaccination schedule, the vaccines will be administered to all participants per Burkina Faso Ministry of Health guidelines, except for 1 group of 50 children aged 15–23 months of age, who will delay MCV-A by 1 month. We anticipate recruiting 250 participants (100 infants aged 9–11 months and 150 children aged 15–23 months) at a study site in Ouagadougou, Burkina Faso. The study duration (recruitment and follow-up) will be approximately 9 months. A trial schedule of events is found in Table 4.

This study is sponsored by the Center for Vaccine Development and Global Health at the University of Maryland School of Medicine in Baltimore, Maryland.

## METHODS

This study will be divided into 2 cohorts, by age, with separate study designs. Cohort 1 will include children 9–11 months of age. Cohort 2 will include children 15–23 months of age. The purpose for our detailed evaluation of safety and immunogenicity is to assess vaccine reactogenicity and the immune response to Vi-TCV and co-administered vaccines. Serum will be collected from all participants on Day 0 (before vaccination) and on post-vaccination Day 28 to quantify anti-Vi and anti-TT antibodies. All children will have an additional 0.5 mL of blood collected on Day 0, before the study vaccination, to test for the presence of malaria parasitemia at baseline. Some studies demonstrate impaired immunogenicity of routine vaccinations in children with *Plasmodiumfalciparum* malaria at sites similar to Ouagadougou [10–12], and we will evaluate this potential effect by measuring for *P. falciparum* parasitemia on Day 0.

Children 9–11 months of age will be eligible for Cohort 1, a double-blind, individually randomized, controlled trial. Up to 100 children will be randomized in a 1:1 ratio to receive Vi-TCV (Group 1) or IPV (Group 2). Participants and parents will not know the study vaccine received. Vi-TCV or IPV will be administered with MR and YFV, as per Burkina Faso’s EPI schedule. These 9–11-month-old children will have immunogenicity to Vi-TCV, YFV, and TT assessed on Days 0 and 28.

Children 15–23 months of age will be eligible for Cohort 2, a randomized safety and immunogenicity study of (1) Vi-TCV when co-administered with routine EPI vaccines (MCV-A and MR) or given alone and (2) MCV-A when co-administered or given alone. Participants in this cohort (up to 150) will be randomized 1:1:1: Group 3 participants will receive Vi-TCV and IPV at Day 0, with a subsequent dose of MCV-A at Day 28; Group 4 will receive MCV-A and Vi-TCV at Day 0; and Group 5 will receive MCV-A and IPV at Day 0. All children will receive MR at Day 0. Cohort 2 will be unblinded on Day 28 for safety and follow-up and to ensure MCV-A receipt in Group 3. These 15–23-month-old children will have serum bactericidal antibodies to *Neisseria meningitidis* serogroup A, anti-Vi antibody, and TT antibody assessed on Days 0 and 28.

We will measure anti-Vi immunoglobulin G (IgG) antibody responses using the VaccZyme Salmonella typhi Vi IgG enzyme-linked immunosorbent assay commercial kit (The Binding Site Group Ltd, Birmingham, UK). Antibody titers will be expressed as enzyme-linked immunosorbent assay units per milliliter (U/mL). Both anti-Vi IgG antibody geometric mean titers and seroconversion, defined as a 4-fold or greater rise in anti-Vi antibody titers from baseline, will be calculated per group. The difference in geometric mean titers between the 2 groups will be compared using a 2-sample Student *t* test on log-transformed titers or a Mann-Whitney U test, as appropriate. The difference in the percentages of participants who seroconverted between the 2 groups will be compared using the Pearson’s chi-square test or Fisher’s exact test, as appropriate.

Participants in both cohorts will have home or clinic visits on Days 3 and 7 following vaccination, for local and systemic adverse event (AE) capture. Non-serious AEs will be assessed up to Day 28. Serious adverse events (SAEs) will be captured throughout the study follow-up.

### Vi-Typhoid Conjugate Vaccine Dose

Typbar-TCV is a Vi-TCV vaccine manufactured by Bharat Biotech International (Hyderabad, India) that consists of 25 μg of Vi polysaccharide conjugated to a nontoxic tetanus toxoid protein carrier. It is given as a 0.5 ml dose by the intramuscular route to infants, toddlers, children, and adults. This vaccine will be shipped from the manufacturer in 5-dose, 2.5 mL vials.

### Inactivated Poliovirus Vaccine Dose

The control vaccine for this study is Inactivated Poliovirus Vaccine (IMOVAX POLIO, Sanofi Pasteur, Lyon, France). This vaccine consists of 40 D-antigen units (DU) of Poliovirus type 1, 8 DU of Poliovirus type 2, and 32 DU of Poliovirus type 3 preserved in 2-phenoxyethanol (5 mg/mL). IPV is given as a 0.5 ml dose by the intramuscular route to infants, toddlers, children, and adults.

### Study Population

Children aged 9–11 months and 15–23 months may enroll, provided they fulfill all inclusion and exclusion criteria (Table 2) and that their parent/guardian completes the informed consent process. The study recruits participants from Schiphra Medical Center in Ouagadougou, Burkina Faso.

**Table 2.** Study Inclusion and Exclusion Criteria

**Inclusion Criteria**	1. A healthy male or female child, either between the ages of 9–11 months or 15–23 months at the time of study vaccination. 2. A child whose parent/guardian resides primarily within the Ouagadougou study area at the time of study vaccinations and who intends to be present in the area for the duration of the trial. 3. A child whose parent/guardian has voluntarily given informed consent.
**Exclusion Criteria**	1. A history of documented hypersensitivity to any vaccine component. 2. Prior receipt of any typhoid vaccine. 3. For children 9–11 months, prior receipt of yellow fever vaccine or any measles-rubella–containing vaccine. 4. For children 15–23 months, prior receipt of meningococcal A conjugate vaccine or a second measles-rubella vaccine. 5. Prior receipt of any measles-rubella–containing vaccine (for children 9–11 months). 6. A history of severe allergic reaction with generalized urticarial, angioedema, or anaphylaxis. 7. A known history of diabetes, tuberculosis, cancer, chronic kidney disease, heart disease, liver disease, a progressive neurological disorder, poorly controlled seizures, or a terminal illness. 8. Severe malnutrition, as determined by mid-upper arm circumference (MUAC) < 12.5 cm. 9. Receipt of any other investigational intervention in the last 6 months or anticipated receipt during the course of the study. 10. Receipt of blood products in the last 6 months. 11. A known human immunodeficiency virus infection or exposure, or other immunosuppressive conditions. 12. Receipt of systemic immunosuppressant or systemic corticosteroids. 13. Any condition determined by the investigator to be likely to interfere with evaluation of the vaccine, to be a significant potential health risk to the child, or to make it unlikely that the child would complete the study.

## STUDY PROCEDURES

### Recruitment and Screening

Following community sensitization using Institutional Review Board (IRB)-approved materials, local community families with children aged 9–11 months and 15–23 months will be given study information in French and/or a local language (including Mossi, Dyula, and Fulani), either individually or in a group setting. The voluntary nature of the study and the right to withdraw will be explained. Parents of eligible children will then be encouraged to ask questions and will provide written informed consent in a semi-private area before enrollment and vaccination. The consent form will have a signature or thumbprint from the child’s parent/guardian before any study procedure. The adult literacy rate in Burkina Faso was 35% in 2014 [13], and providing a thumbprint is a locally acceptable equivalent for a written signature. A copy will be given to the parent/guardian and another copy will be filed on site. If a parent/guardian is unable to read or sign, an impartial witness will document that all information was provided and consent was freely given. After informed consent is obtained, each potential participant will be screened for eligibility, according to the inclusion/exclusion criteria.

### Randomization

Randomization will be computer-generated to allocate eligible participants in a 1:1 ratio for the 9–11-month-old cohort or a 1:1:1 ratio for the 15–23-month-old cohort, using stratified block randomization with varying block sizes from 6–12.

### Blinding

Vi-TCV and IPV have similar pharmaceutical presentations. Unblinded study personnel will be responsible for vaccine preparation and administration. These unblinded personnel will not be involved in study-related assessments or have contact with subjects for the purpose of data collection following the study vaccination. Study participants, their family members, and study staff will not know the assigned vaccine group. The 15–23-month-old cohort will be unblinded on Day 28, so that Group 3 can receive MCV-A at this visit.

### Enrollment

**Figure 1.**
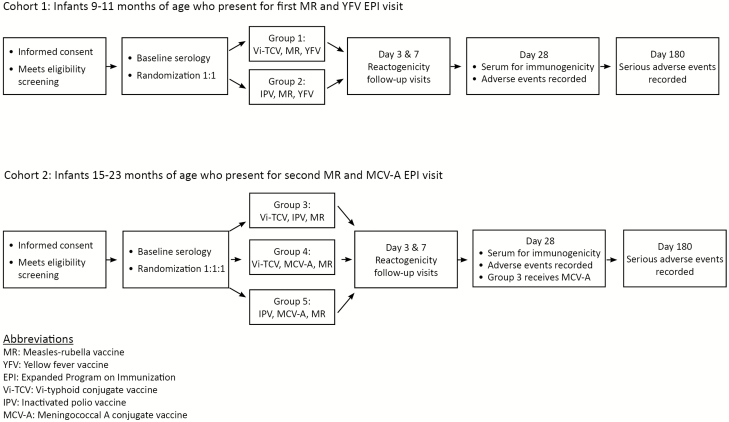
Trial flow diagram. EPI, Expanded Program on Immunization; IPV, inactivated poliovirus vaccine; MCV-A, meningococcal A conjugate vaccine; MR, measles-rubella vaccine; Vi-TCV, Vi-typhoid conjugate vaccine; YFV, yellow fever vaccine.

Screening and enrollment visits may occur on the same day. Study staff will review the participant’s eligibility criteria, medical history, concomitant medications, and vaccination history. All participants will have their temperature taken and 3–5 mL of blood drawn for baseline immunogenicity and *P. falciparum* malaria parasitemia testing. Participants will be administered both the study and routine vaccinations (Figure 1), then asked to remain in the study area for at least 30 minutes in case of a serious reaction. Any immediate reactions will be assessed and recorded.

### Follow-up

Follow-up visits may be either routine or unscheduled. Routine visits will occur on Days 3, 7, 28, and 180. On Days 3 and 7 following vaccination, participants will be asked to return to the study clinic to assess reactogenicity. During these visits, solicited and unsolicited AEs will be recorded. Alternatively, these visits may be conducted in the home. On Day 28 following vaccination, participants will be asked to return to the study clinic for a follow-up visit. During this visit, the treatment groups in the 15–23-month-old cohort will be unblinded, participants will have approximately 3–5 mL of blood drawn to measure the Vi antibody responses for typhoid (all age groups), measure the TT serum IgG antibody levels (all age groups), and conduct the yellow fever plaque reduction neutralization testing (9–11-month-old group only) or measure the meningococcal A serum bactericidal antibody titers (15–23-month-old group only), which will be used for the primary and secondary objectives, as listed. Participants will have their final visit on Day 180, when interim SAEs will be recorded.

During unscheduled visits, participants will undergo standard procedures for the evaluation and treatment of illness, as defined by the Ministry of Health. Illnesses and other medical issues will be documented as AEs in participant records, and their seriousness, severity, relationship to the study product, and expectedness will be recorded.

## ADVERSE EVENTS

### Immediate Reactions

All participants will be observed for immediate reactions for 30 minutes after vaccination. Immediate reactions will be assessed by a study physician or appropriately trained medical staff member, and recorded on the appropriate case report form (CRF).

### Solicited Local and Systemic Reactions

On Day 3, participants will be assessed for local and systemic reactions and will be asked about any events after vaccination on Day 0 and any current symptoms; on Day 7, participants will be assessed for local and systemic reactions and will be asked about current symptoms. Using a standardized data collection instrument, the following local and systemic reactions will be documented and graded on predefined scales, based on a functional assessment or the magnitude of the reaction: fever, injection site pain/tenderness, swelling, erythema, and irritability.

### Note on Timing

All solicited and unsolicited AEs occurring through Day 7 post-vaccination will be assessed and recorded. All AEs that meet the criteria for an SAE and that occur through 6 months post-vaccination will be assessed and recorded.

### Definition of Adverse Event

An AE is any untoward medical occurrence in a participant to whom Vi-TCV or IPV has been administered, including occurrences that are and are not caused by or related to the vaccine. All AEs related to study vaccination that are reported by telephone or by home or clinic visits in the first 7 days after vaccination will be recorded, including the event description, the date of onset, the resolution, the level of severity, an assessment of relatedness to the study, and the outcome.

### Seriousness of Event

An untoward medical occurrence that meets any one of the following criteria is considered serious: results in death; is life-threatening; requires inpatient hospitalization or a prolongation of an existing hospitalization; or results in a persistent or significant disability. Other important medical events may be considered serious if the event jeopardizes the participant or requires intervention to prevent 1 of the above consequences.

### Protocol Deviations

Protocol deviations will be collected in a CRF and kept in the study record. Deviations that affect the study’s scientific integrity or a volunteer’s rights/safety will be reported to the University of Maryland and local IRBs.

### Analysis

Study hypotheses and power analyses are found in Table 3. The intention-to-treat population includes all randomized individuals, in the vaccine groups to which they were assigned, and all follow-up time, beginning with the time of randomization. The primary analysis will be based on this population. The per-protocol population includes all vaccinated individuals, in the vaccine groups based on the vaccines they received. Additional details of study analyses will be included in a statistical analysis plan, which will be written by the study biostatistician before the study’s data analysis begins.

**Table 3.** Study Hypotheses and Power Analyses.

	Hypothesis	Power Analysis
**Cohort 1:** Children 9–11 months of age (n = 100), enrolled in a double-blind, individually randomized, controlled trial. With an expected 10% dropout rate, we anticipate 90 children (90% of 100 enrolled) will complete follow-up assessments: 45 in Group 1 and 45 in Group 2.	Reactogenicity following Vi-TCV given together with YFV and MR (Group 1) is not significantly different from reactogenicity following IPV given together with YFV and MR (Group 2).	The expected incidence of any grade 2 or greater solicited, systemic adverse events over 7 days following vaccination is 10% in Group 2 [14]. Sample sizes of 45 in Group 1 and 45 in Group 2 achieve 80% power to detect a minimum difference of 0.23 (= 33% - 10%), using a 2-sided z-test with unpooled variance and a significance level of 5% (PASS 15).
	The percentage of participants with seroconversion (≥2-fold rise) in yellow fever neutralizing antibodies following a single dose of Vi-TCV given together with YFV and MR (Group 1) is not significantly different from the percentage of participants with seroconversion (≥2-fold rise) in yellow fever neutralizing antibodies following a single dose of IPV given together with YF and MR (Group 2).	In Group 2, the expected seroconversion proportion is 70% [14]. Sample sizes of 45 in Group 1 and 45 in Group 2 achieve 80% power to detect a minimum difference of 0.28 (= 70% - 42%), using a 2-sided z-test with unpooled variance and a significance level of 5% (PASS 15).
**Cohort 2:** Children 15–23 months of age (n = 150) will be randomized 1:1:1 to 1 of 3 treatment groups. With an expected 10% dropout rate, we anticipate 135 children (90% of 150 enrolled) will complete follow-up assessments: 45 in Group 3, 45 in Group 4, and 45 in Group 5.	Reactogenicity following a single dose of Vi-TCV given together with IPV and MR (Group 3) is not significantly different from reactogenicity following a single dose of Vi-TCV given together with MCV-A and MR (Group 4).	The expected incidence of any grade 2 or greater solicited, systemic adverse events over 7 days following vaccination is 10% in Group 3 [14]. Sample sizes of 45 in Group 3 and 45 in Group 4 achieve 80% power to detect a minimum difference of 0.23 (= 33% - 10%), using a 2-sided z-test with unpooled variance and a significance level of 5% (PASS 15).
	Reactogenicity following a single dose of Vi-TCV given together with MCV-A and MR (Group 4) is not significantly different from reactogenicity following a single dose of IPV given together with MCV-A and MR (Group 5).	The expected incidence of any grade 2 or greater solicited systemic adverse events over 7 days following vaccination is 10% in Group 5 [14]. Sample sizes of 45 in Group 4 and 45 in Group 5 achieve 80% power to detect a minimum difference of 0.23 (= 33% - 10%), using a 2-sided z-test with unpooled variance and a significance level of 5% (PASS 15).
	The percentage of participants with seroconversion (≥4-fold rise) in meningococcal A bactericidal antibodies following a single dose of Vi-TCV given together with MCV-A and MR (Group 4) is not significantly different from the percentage of participants with seroconversion (≥4-fold rise) in meningococcal A bactericidal antibodies following a single dose of IPV given together with MCV-A and MR (Group 5).	In Group 5, the expected seroconversion proportion is 90% [14]. Sample sizes of 45 in Group 4 and 45 in Group 5 achieve 80% power to detect a minimum difference of 0.23 (= 90% - 67%), using a 2-sided z-test with unpooled variance and a significance level of 5% (PASS 15).
	The percentage of participants with seroconversion (≥4-fold rise) in Anti-Vi IgG antibodies following a single dose of Vi-TCV given together with IPV and MR (Group 3) is not significantly different from the percentage of participants with seroconversion (≥4-fold rise) in Anti-Vi IgG antibodies following a single dose of Vi-TCV given together with MCV-A and MR (Group 4).	In Group 3, the expected seroconversion proportion is 95% [8]. Sample sizes of 45 in Group 3 and 45 in Group 4 achieve 80% power to detect a minimum difference of 0.20 (= 95% - 75%), using a 2-sided z-test with unpooled variance and a significance level of 5% (PASS 15).

Abbreviations: IgG, immunoglobulin G; IPV, inactivated poliovirus vaccine; MCV-A, meningococcal A conjugate vaccine; MR, measles-rubella vaccine; Vi-TCV, Vi-typhoid conjugate vaccine; YFV, yellow fever vaccine.

### Ethical Considerations

The study will be conducted in accordance with the principles of the Declaration of Helsinki and in accordance with the principles of good clinical practice. The protocol and informed consent will be submitted to the University of Maryland IRB, the Regulatory Authority of the Ministry of Health of Burkina Faso, and the Ethics Committee for Health Research in Burkina Faso for written approval. Reports will be provided to the University of Maryland IRB and other parties annually. To compensate for their time and travel related to study activities, the participant’s parent/guardian will receive $15 (7500 Franc Communauté Financière Africaine) for every scheduled clinic visit.

To protect participant confidentiality, study records will be stored in a locked office or in a password-protected, secure database. No information concerning the study or the data will be released to any unauthorized third party.

### Potential Risks of Vi-Typhoid Conjugate Vaccine

In clinical trials among Indian children [8], adverse reactions were predominantly minor and transient. Local reactions, such as injection site pain, erythema, and induration, usually resolved within 48 hours of vaccination. Fevers ≥38°C have been observed in approximately 1–4% of vaccinees [15]. A post-licensure study of Vi-TCV co-administration with a measles-rubella–containing vaccine was conducted, as was a Phase IV comparator study with the WHO pre-qualified Vi-polysaccharide vaccine. In both studies, no safety signals were reported and the Vi-TCV safety profile was comparable to the respective comparator. Post-marketing surveillance of Vi-TCV, based on approximately 3000 reports, has shown fever, pain, and swelling around the injection site to occur in 1–10% of vaccinees in any age group, with no SAEs reported [16]. The WHO’s Global Advisory Committee on Vaccine Safety review found no safety signal related to the vaccine [16].

### Potential Risks of Inactivated Poliovirus Vaccine

The most common reactions following the receipt of an IPV are local injection site reactions (eg, pain, erythema, and induration) and moderate, transient fever. Other rare reactions (less than 0.01% of vaccine recipients) include edema, lymphadenopathy, transient arthralgia and myalgia, convulsions, headaches, paresthesia, somnolence, irritability, and allergic reactions (urticarial, facial edema, or anaphylactic shock) [17].

### Data

Research records will be stored in a locked room and on a secure electronic database, in compliance with International Conference for Harmonisation E6. Only authorized personnel will have access to these data. Regulatory agencies may examine the records for quality assurance reviews, audits, and the evaluation of the study’s safety and progress. Safety and immunogenicity data will be recorded on paper CRFs for subsequent data entry or entered directly into an electronic database. Study-specific CRFs will be generated.

### Quality Control and Quality Assurance

The site developed a protocol-specific quality management plan in conjunction with the University of Maryland Center for Vaccine Development and Global Health Office of Regulatory Affairs and Quality Management. This plan details how data will be evaluated for protocol compliance, which documentation will be reviewed, and the methods for training staff.

### Monitoring

The sponsor’s team will visit the site before the study’s start to discuss the protocol and data collection procedures with site personnel. Before participant enrollment, required regulatory documents must be available. Subsequent study monitoring will be conducted according to the sponsor’s requirements. Study monitors will periodically review the progress and access all records necessary to ensure the ethical and safe study conduct and the integrity/validity of recorded data.

### Plans for Dissemination of Results

The principal investigator and sponsor investigator will oversee the collection, approvals, and dissemination of study data. All publications and presentations will be reviewed by each co-investigator before submission. In accordance with the Bill & Melinda Gates Foundation policy, all study-related publications will be open access. Authorship will comply with International Committee of Medical Journal Editors guidelines.

### Clinical Trial Coordination

An on-site study physician will manage the daily study activities, with visits twice weekly by a senior study physician. Local study teams will participate in weekly teleconferences with international investigators to discuss the study’s progress, challenges, and planning. International investigators make regular visits to the study sites approximately every other month to review the progress and assist in study coordination.

The study is anticipated to begin recruitment activities in late 2018.

## DISCUSSION

This randomized, controlled clinical trial among infants and young children in Ouagadougou, Burkina Faso, will be the first in West Africa to test the pre-qualified Typbar-TCV. The study will provide the first information on co-administration of this vaccine with YFV and MCV-A, as recommended by the WHO. The rationale for the 3 arms in the older children is that both MCV-A and TCV contain tetanus toxoid as the carrier protein: thus, our study design allows us to carefully assess reactogenicity when those vaccines are given alone or together, and evaluate any effect on the antibody responses that might occur with a shared carrier protein. Such a phenomenon was noted with *Haemophilus Influenzae* Type b vaccines [18] and is thought to occur due to interference by a common protein carrier with common immunogenic epitopes [19]. We did not include an assessment of measles and rubella antibody responses, since this has been previously tested and we have attempted to minimize blood collection volumes in these young children.

Malaria parasitemia is prevalent among young children in Burkina Faso. We will test for *P. falciparum* parasitemia at Day 0 to evaluate the potential effect on the immunogenicity of Typbar-TCV and of the other vaccines administered.

**Table 4.** Trial Schedule of Events. Abbreviations: AE, adverse event; SAE, serious adverse event.

Study Visit Type	Recruitment	Screening	Eligibility, enrollment, vaccination	Follow up	Follow up	Follow up	Follow up	Early termination
Study Visit Number		V00	V01	V02	V03	V04	V05	
Study Time point			D0	D3	D7±1	D28±3	D180±7	
Study introduction, recruiting	X	X^a^	X^a^					
Obtain informed consent		X	X^a,b^					
Review eligibility criteria		X	X^a,c^					
Review medical history		X	X^a,c^	X^c^	X^c^	X^c^	X^c^	X^c^
Review concomitant meds		X	X^a,c^	X^c^	X^c^	X^c^	X^c^	X^c^
Review vaccination history		X	X^a,c^	X^c^	X^c^	X^c^		X^c^
Axillary temperature^d^		X	X^e^			X		X
Height and weight and Middle Upper Arm Circumference (MUAC)		X	X^e^				X	X
Targeted physical exam		X^˚^	X^a,c,e,˚^			X^c,˚^		X^c,˚^
Vaccination			X			X^f^		
30-min post-vaccination evaluation			X					
Post-vaccination reactogenicity assessment				X	X			X
Venous blood collection for immunogenicity			X^e,g^			Xg		X^g^
Venous blood collection for malaria parasitemia			X^e^					
Assess and record SAES			X^h^	X	X	X	X	X
Assess and record solicited AEs			X^h^	X	X			X^i^

^a^If not performed at previous visit.

^b^Confirm informed consent for study procedures.

^c^Review/confirm information or activity in participants previously consented and screened.

^d^Participants must not eat or drink anything hot or cold within 10 minutes before taking oral temperature.

^e^Prior to study vaccination and used as a baseline.

^˚^Targeted physical exam if indicated based on interim medical history.

^f^Only for children in Group 3 in the 15 through 23 month old cohort

^g^3-5m1 of blood collected for immunogenicity and noninterference testing ^h^If AE/SAE occurs post-vaccination

^i^Only if visit occurs within 7 days after study vaccination.

## Notes

***Author contributions.*** K. M. N., K. L. K., S. B. S., E. T. R., and M. B. L. conceived this study, developed the protocol, developed the standard operating procedures, managed the ethical submissions, and reviewed manuscript. Y. L. developed the statistical analysis plan and reviewed the manuscript. All authors read and approved the final manuscript.

***Acknowledgments.*** The authors thank the Bill & Melinda Gates Foundation and the staff of Schiphra Medical Center and Groupe de Recherche Action en Santé in Ouagadougou.

***Financial support.*** This publication is based on research funded in part by a grant from the Bill & Melinda Gates Foundation (OPP1151153).

***Supplement sponsorship.*** This supplement is sponsored by the Center for Vaccine Development and Global Health (CVD) at the University of Maryland School of Medicine.

***Potential conflicts of interest.*** K. M. N. is a member of the World Health Organization Strategic Advisory Group of Experts on Immunization. All other authors report no potential conflicts. All authors have submitted the ICMJE Form for Disclosure of Potential Conflicts of Interest. Conflicts that the editors consider relevant to the content of the manuscript have been disclosed.
